# Unravelling the Influence of Extraction Techniques on Protein Yield and Nutritional Value in Lesser Mealworm Larvae †

**DOI:** 10.3390/molecules29174220

**Published:** 2024-09-05

**Authors:** Andrea Fuso, Giulia Leni, Augusta Caligiani

**Affiliations:** 1 Department of Food and Drug, University of Parma, Parco Area Delle Scienze, 27/A, 43124 Parma, Italy; andrea.fuso@unipr.it (A.F.); augusta.caligiani@unipr.it (A.C.); 2 Department for Sustainable Food Process, Università Cattolica del Sacro Cuore, Via Emilia Parmense 84, 29122 Piacenza, Italy

**Keywords:** *Alphitobius diaperinus*, insect, novel protein, protein quality, amino acid

## Abstract

In the present work, chemical and enzymatic assisted techniques were compared for protein extraction from lesser mealworm larvae (LM, *Alphitobius diaperinus*), recently approved as a novel food in the European Union. All extracts showed appreciable nutritional quality, with quantities of essential amino acids above the reference standard. Conventional alkali extraction allowed the isolation of only 73% of the protein, preserving the amino acid composition but potentially causing denaturation or racemisation. The “stepwise” method, following the Osborne fractionation, improved protein recovery to 91% by isolating four fractions with different solubility properties. Additionally, enzymatic hydrolysis using *Bacillus licheniformis* proteases was also tested, and it provided hydrolysates with an average degree of hydrolysis of 14%, making them a potential hypoallergenic solution. Overall, these findings indicate the ability to tailor the composition of LM protein to meet specific needs, offering promising prospects for the use of insect protein ingredients in various applications.

## 1. Introduction

Edible insects are currently consumed as a traditional food in many regions of Asia, South America, and Africa, while in Europe and North America they are considered a new, promising alternative to conventional animal sources [1]. In fact, due to the future increase in world population and meat consumption, the finding of alternative animal proteins has become of primary interest, with edible insects being considered as a solution. As a matter of fact, the industrial production of insects has a wide range of environmental advantages if compared with livestock production: lower greenhouse gas emissions, less use of land and water, and higher efficiency in feed conversion [2]. Furthermore, insects can grow on a large spectrum of organic material, food waste included, perfectly meeting the circular economy perspective [3].

Among the more than 2000 insect species consumed as food worldwide, only some of them are authorised by the European Union (EU). Until now, four insect species have been authorised by the European Commission as novel foods: lesser mealworm (LM, *Alphitobius diaperinus*), house cricket (*Acheta domesticus*), yellow mealworm (*Tenebrio molitor*), and migratory locust *(Locusta migratoria*) [3]. Besides these, many other applications are currently under evaluation by the European Food Safety Authority (EFSA).

Apart from being a potentially good source of fat, minerals, vitamins [4], and chitin [5], edible insects present a notable quantity of proteins, which ranges from 13% to 77%, with a high content of essential amino acids that makes them nutritionally relevant for humans and animals [6]. Although all the environmental and nutritional advantages are well known, western consumers are not perfectly acquainted with insect consumption since they are not part of their historical food tradition [7]. However, it has been demonstrated how insect acceptability can be increased by processing them into ingredients that can be thereafter incorporated into more familiar food products [8]. The Global Market Insights Report estimated that the edible insects market will grow by 47% between 2019 and 2026, and the protein bar/protein supplements segment is anticipated to hold significant market share due to the growing demand for high-quality alternative protein sources, especially for athletes and the elderly population [9]. Indeed, proteins might be reckoned the most intriguing macronutrient in insects thanks to their nutritional quality, and therefore, their isolation from this matrix must be considered as an approach. In this scenario, it is mandatory to design optimum extraction protocols allowing to produce insect protein ingredients with a good balance between extraction yield and protein quality.

Insects’ separation into fractions is usually performed through wet or dry fractionation [10]. Usually, protein extraction via wet fractionation includes protein solubilisation in alkaline conditions (pH 8–10) and their following precipitation at the isoelectric point [11]. Nevertheless, alkali extraction, while typically effective, is recognised for its potential to induce reactions within the protein structure. These reactions may include denaturation, hydrolysis, and racemization, as well as the formation of compounds like lysinoalanine and other cross-linked substances, ultimately diminishing the nutritional quality. The mildest extraction methods, based on neutral aqueous or hydroalcoholic systems or on proteolytic enzymes, have been previously tested on black soldier fly (*Hermetia illucens*) [12]. In addition, the protein yield of extraction can be enhanced by additional sonication treatment and/or the application of enzymes [13]. Regarding proteins from LM, only a limited number of studies have been performed with the attempt to isolate and obtain high yields of protein ingredients. Some works have already demonstrated the feasibility of producing protein ingredients from LM by performing both chemical [14] and enzymatic [15,16] extractions. LM proteins were also extracted by alkali to be used as emulsifiers [17], and in other cases the soluble protein fraction was isolated to study its nutritional value, stability versus enzymatic activities, and digestibility [18]. The aqueous extraction methods, albeit easy and feasible to apply, generally give relatively low yields. 

Given the increasing interest in edible insects as a sustainable protein source and the diverse extraction methods available, we hypothesised that different extraction protocols will yield varying protein fractions from LM larvae. Furthermore, we predict that these protein fractions will exhibit differences in nutritional composition and functional properties, thus influencing their potential applications in the food industry. In this direction, in the present work, a distinct emphasis was placed on elucidating the most effective methods for extracting proteins from LM larvae. The investigation encompassed a comprehensive analysis of chemical and enzymatic protocols, meticulously tailored to ensure maximal protein recovery while preserving nutritional integrity. For the first time, LM proteins extracted by different methodologies have been compared for their extraction yield and amino acid composition, providing important insight regarding the optimal LM protein extraction protocols for future applications in the food sector.

## 2. Results and Discussion

In this work, different extraction protocols were evaluated, and the composition of LM protein fractions was studied in terms of protein yield. Also, the nutritional value was assessed by analysing the amino acid composition of the whole insect and of each fraction obtained from the different protocols tested. The determination of total amino acid composition represents the benchmark for insect protein characterisation. Firstly, because it is recognised that the extraction process can affect the amino acid distribution of insects’ proteins [19], but also to determine the correct amount of proteins extracted. The protein content is generally calculated from total nitrogen using a standard nitrogen-to-protein conversion factor of 6.25 [20]. However, the nitrogen content in LM (and in insects in general) includes nitrogen originating from non-protein sources as well, namely from chitin, a polymer of N-acetylglucosamine composing the insect exoskeleton, making necessary the determination of amino acids for the accurate protein content assessment [21].

### 2.1. Total Amino Acids in Whole Lesser Mealworm Larvae 

The results of the amino acid analysis related to whole LM larvae are reported in Table 1, where the essential amino acid content is also compared with reference proteins. 

The total amount of amino acids was determined as 58.9 ± 0.1% DM, corresponding to a global protein content equal to 51% DM. From this amino acid composition, it was possible to determine the nitrogen-to-protein conversion factor, which turned out to be 5.74 ± 0.01, thus quite far from the widely used 6.25 and in agreement with that of Janssen et al. (2017). Glutamic acid plus glutamine and aspartic acid plus asparagine turned out to be the most abundant amino acids (7.74% and 5.42% DM, respectively). On the contrary, tryptophan and methionine were the least abundant by far, with quantities around 1.2% DM. In general, the amino acid distribution is in line with that found in other works in the literature for LM and other insect species [23]. In a previous work of ours, very similar results were obtained when comparing insects’ and conventional protein sources’ amino acid patterns studying another insect species, namely black soldier fly, even though in that case lysine was quite affected by different diets, thus resulting sometimes definable as the limiting amino acid [24]. 

Regarding protein quality, the essential amino acid content (reported as mg/g protein) was compared with the amino acid pattern of egg white and soybean, which are two valuable and common protein sources in the human diet. The protein composition of LM showed higher levels of histidine, tryptophan, phenylalanine, and tyrosine, with slightly lower quantities of leucine and isoleucine observed, along with notably reduced levels of sulphur-containing amino acids. Additionally, LM exhibited comparable levels of threonine, valine, and lysine. Comparative analysis against the amino acid requirements for adult humans as outlined by FAO revealed that LM proteins surpassed these requirements for all essential amino acids, indicating none were found to be limiting.

### 2.2. Lesser Mealworm Protein Extractions

The amino acid analysis demonstrated how LM represents a valuable source of alternative proteins for future human consumption. However, introducing insects into the eating habits of EU consumers is not easy, and the current market strategy is to develop highly processed insect-based foods familiar in Western diets, such as bread, biscuits, and pasta, or to use insect protein-rich ingredients in food supplements or enriched foods, in which the insect is hidden. The logical consequence of this trend is that insects need to undergo many different technological processing steps, and, in the specific case of protein extraction and purification, the extraction technology used directly impacts protein composition and nutritional quality. To gain insights into the effect of extraction on LM protein quality, different protocols have been performed after a defatting step, which allowed to obtain a starting biomass with 59.7% of residual protein. The extraction protocols applied in this work include both chemical and enzymatic procedures.

As per chemical protocols, an alkali treatment, also called “one shot” extraction, was applied and compared to the Osborne fractionation, also called “stepwise” extraction, and commonly used for cereals. Besides the chemical methods, enzymatic hydrolysis was also performed as a valuable green alternative to minimise the use of chemical reagents and, therefore, the environmental and economic impacts. Moreover, the use of proteases triggers protein chain degradation, increasing protein digestibility, and releasing bioactive and potentially hypoallergenic peptides [25]. The latter factor is not of secondary importance if we consider the allergenicity properties of LM and other insect species and thus the potential risk for future consumers [3]. At the end of all these extractions, a centrifugation step allowed the separation of extracted proteins from the insoluble pellet, which is supposed to be mainly characterised by the presence of non-solubilised protein and chitin [12]. The protein extraction yields of the different protocols are reported below in Table 2. 

The extractions with alkali (“one shot”) and with protease allowed to achieve a statistically equivalent and satisfactory protein dissolution, equal to 73% and 76% of the total proteins, respectively. These yields, however, are significantly lower than that achieved with the “stepwise” fractionation that allowed to globally extract 91.3% of the total protein. 

The “stepwise” extraction led to four protein fractions having different solubility properties. The first fraction represented water-soluble proteins (albumins), the second fraction constituted proteins soluble in saline solution (globulins), and the third and fourth fractions represented alcohol- and alkali-soluble proteins (prolamins and glutelins, respectively). The solid residue obtained after the four extraction steps represented the chitin fraction with the residual non-soluble proteins. 

In general, from a quantitative point of view, the extraction yield obtained in this work perfectly agrees with a previous one performed on black soldier fly, in which a 91% yield was gained using the same “stepwise” method [12]. Specifically, the most protein-rich fraction was the one named glutelin, accounting for 52.4% of the total protein. Albumin extract contained 30.8% of the total initial protein, whereas globulin and prolamin were instead rather scarce, comprising as a sum a total protein percentage of about 8%. These results suggest that most LM proteins are either extractable in water or alkali solution. The latter, in particular, has been used in different works [17,26], even though different solutions, such as citric acid/disodium phosphate buffer, were considered by other authors [20]. However, our results suggest that alkali extraction by itself is not sufficient to achieve such a high yield. It is interesting to note that if on the one hand alkali extraction allowed to achieve 73% of proteins, on the other hand, the sum of albumins and glutelins corresponded to a significantly higher yield, namely 83%. These results are surprising since a 2 h extraction with alkali at 50 °C is supposed to have greater efficiency than a method consisting of water for 1 h at 4 °C + alkali for 1 h at 4 °C. The reason might be found in the residual presence of sodium chloride (coming from the previous step for globulin isolation) in the alkali solution during the last part of the “stepwise” experiment. Indeed, it has been recently outlined how a salting-in-based approach using NaCl and NaOH together leads to an increase in extraction efficiency [27]. 

The chemical techniques were additionally evaluated against protease-assisted extraction. The protease derived from *Bacillus licheniformis* demonstrated the capability to dissolve approximately 76% of the total proteins, a marginally superior yield compared to that achieved by Caligiani et al. under analogous conditions with black soldier fly prepupae [12]. The enzyme therefore had a positive action on the extraction of LM proteins, thanks to the hydrolysis of the peptide bonds and the formation of more soluble oligopeptides and free amino acids. Although the enzymatic extraction yield was lower than that observed for chemical stepwise extraction, it is important to note that the enzymatic method may reduce allergenic risk by releasing potentially hypoallergenic oligopeptides [28]. However, this potential was not directly evaluated in the current study and requires further investigation.

### 2.3. Characterisation of LM Extracts

#### 2.3.1. Total Amino Acids

All the extracts were also characterised in terms of protein quality in order to evaluate their potential nutritional value and specific changes in protein composition with respect to the whole LM protein. The complete amino acid profile of protein fractions was reported as a relative distribution in Table S1 of the Supplementary Material, whereas the evaluation of the amino acid score of each LM fraction (compared to egg white) is reported below in Table 3.

Small variations were identified in the amino acid score profile of LM before and after the different extraction protocols. Among the different extracts, the prolamin fraction and the enzymatic hydrolysate presented an averagely higher amino acid score compared to LM larvae (1.11 and 1.10 vs. 1.09). On the contrary, the amino acid score of the other fractions slightly decreased, albeit remaining of great quality, ranging from 0.90 in the albumin extract to 1.04 in the “one shot” alkali extraction. These findings indicate that the methods used for extraction did not impact the nutritional quality of LM proteins in relation to their essential amino acid content. Methionine emerged as the primary limiting amino acid in all extracts except for the prolamin fraction, where isoleucine took on that role. This result confirms once again the low content of sulphurated amino acids in LM larvae, as already reported in Table 1. However, to the best of our knowledge, this is the first time that such an investigation has been carried out on protein fractions isolated from LM larvae. Nevertheless, by individually analysing each amino acid score, it was found that histidine resulted in the most affected amino acid, with a score varying between 1.03 in the enzymatic extract and 3.22 in the prolamin extract, compared to 1.82 calculated in the LM starting material.

It is important to note that the protein quality is not the only parameter to consider since protein extracts are often supposed to be used for their techno-functional proteins. Indeed, it is widely accepted that insect proteins are promising biomolecules for their gelling, foaming, emulsifying, and water- and oil-holding capacities, and insect processing can affect them [11]. Considering the total amino acid profile (Table S1), it was found that the ‘one shot’ NaOH extract, despite the lower yield, was the most similar to the whole LM protein in terms of amino acid composition, suggesting no selectivity of the hot alkali extract with respect to specific classes of protein. A strict similarity is also displayed for the enzymatic hydrolysate, and this is not surprising due to the low selectivity of the enzyme regarding the protein hydrolysed and solubilized. Interestingly, significant differences are displayed in the amino acid composition of the different protein extracts obtained by the stepwise method (Table S1). With respect to the whole LM protein, the albumin fraction is particularly rich in glutamic acid/glutamine and proline and poor in phenylalanine, arginine, and tyrosine. Globulin and glutelin fractions showed abundant quantities of acidic amino acids (both aspartic and glutamic acids), while the prolamin fraction contained basic amino acids (arginine, histidine, and lysine). These different amino acid patterns, and especially the different protein charge due to the acidic and basic amino acids, suggest also different functionality and technological properties that merit further investigation. In fact, high protein charge is essential for foaming and emulsifying capacity due to high electrostatic repulsion between charged adsorbed proteins to the interface [29]. The low tyrosine content in the albumin extract might also limit browning reactions, which have been demonstrated to occur as a consequence of melanisation in insects, thus improving consumer acceptability [30]. Additionally, the high proline content may confer thermal stability to this fraction [31]. Moreover, since all the extraction steps were made in mild conditions, the proteins collected were supposed to be intact, and this represents indeed the best method to isolate proteins to be used for high-added-value products (e.g., food supplements).

However, whether they are used for technological or nutritional purposes, insect protein extract relates to another issue, namely allergenicity risk. In fact, the possibility of cross-reactivity and co-sensitisation between edible insects, crustaceans, and house dust mites has been deeply discussed [32]. Hall and colleagues reported that cricket proteins must be hydrolysed to a degree of at least 60% to totally extinguish the IgE response to tropomyosin, whereas a degree of hydrolysis of 52% showed similar reactivity to the non-hydrolysed protein [33]. In our previous work, it was demonstrated that a DH% of 22% was sufficient to eliminate the reactivity of LM protein with the IgE from patients allergic to crustaceans [28].

As enzymatic extraction by the protease from *Bacillus licheniformis* stands out as the method among those employed here that enables the hydrolysis of insect protein into potentially hypoallergenic oligopeptides, this extract was additionally assessed for its DH% and the liberation of free amino acids.

#### 2.3.2. Degree of Hydrolysis and Free Amino Acids of Enzymatic Hydrolysate

**Figure 1:**
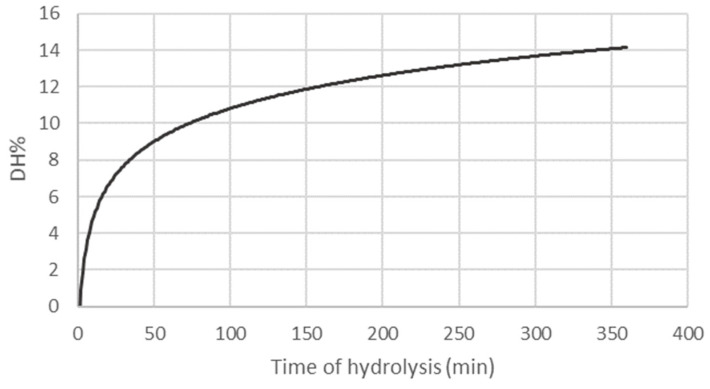
Kinetics of enzymatic hydrolysis performed with protease from *Bacillus licheniformis* on LM. The results describe the degree of hydrolysis (DH%) variations according to the time of hydrolysis (min) and are the means of three separate extractions.

In this work, DH% was calculated at each point of the enzymatic reaction by a pH-STAT system, and the kinetic curve is reported in Figure 1.

The DH was calculated as 14 ± 1% after 6 h of hydrolysis, and the kinetic curve underlined the proximity to the plateau phase. By the inverse formula of DH% (Equation (1)), it was possible to calculate the average length of peptides composing the hydrolysate, which resulted in being made of seven amino acids. According to Nagodawithana and colleagues, the length of peptides can impact the allergenic properties of protein hydrolysates. They suggest that keeping the average molecular weight below 1500 Da might decrease the allergenic potential of a food product [34]. Given an estimated average molecular mass of 110 Da for residual amino acids, it suggests that the hydrolysates in question might qualify as hypoallergenic. However, further in vitro and in vivo experiments are imperative to validate this proposition. 

Proteolytic enzymes demonstrate their efficacy not just by liberating oligopeptides but also by releasing free amino acids. This directly enhances the digestibility of the protein extract. Consequently, the composition of free amino acids in the enzymatic hydrolysate was analysed using UPLC/ESI-MS and compared to the original free amino acid profile in the raw material prior to extraction (Table 4). 

As expected, the free amino acid content significantly increased after the enzymatic hydrolysis, moving from 6.4 ± 0.6 mg/g DM in LM as such to 166.7 ± 4.1 mg/g DM in the enzymatic hydrolysate. In particular, the amount of free amino acid after enzymatic hydrolysis accounted for 1.5 ± 0.3% of the supernatant extracted, thus representing 29% of total extracted proteins (results obtained from total amino acid analysis). These data were in line with the ones reported by Leni et al. [35], where LM subjected to enzymatic hydrolysis with the protease from *Bacillus licheniformis* released 25% of total amino acids in the form of free amino acids. This result is particularly interesting if we consider that in the current work, a comparable amount of free amino acid was extracted by reducing the time of hydrolysis and controlling the reaction by a pH-STAT system. By deeply analysing the free amino acid profile of enzymatic hydrolysate, glutamic acid resulted in the most abundant free amino acid detected (22.0 mg/g DM), followed by alanine, leucine, and lysine (19.9 mg/g DM, 17.6 mg/g DM, and 17.5 mg/g DM, respectively). On the contrary, histidine and glutamine were identified as the less abundant free amino acids released after the proteolysis performed by the protease from *Bacillus licheniformis*. The concentration of the free amino acids, together with their threshold values, significantly influences the taste profile of the protein hydrolysate [36]. Understanding how they impact the hydrolysate’s sensory attributes plays a crucial role in formulation. As an example, the high level of glutamic acid, known for its role in evoking the umami taste sensation, likely contributes to the rich and full-bodied taste of the hydrolysate. It is clear that the taste of protein hydrolysate is a result of the intricate interplay between various free amino acids. 

Indeed, the abundant presence of oligopeptides and readily absorbable essential amino acids, surpassing intact proteins in digestibility, not only reduces the likelihood of allergies but also boosts the nutritional quality of insect hydrolysates intended for both feed and food applications. Furthermore, the presence of multiple amino acids with different taste characteristics can create a complex taste profile, offering a well-rounded and appealing sensory experience. In addition, proteases have been shown to release encrypted bioactive peptides present within the insect proteome, exhibiting a spectrum of diverse bioactivities, including but not limited to antioxidant, anti-angiotensin-converting enzyme, and anti-dipeptidyl peptidase-IV [37,38]. Consequently, the integration of insect-derived protein hydrolysates into food formulations emerges as a promising avenue for the development of functional novel foods.

## 3. Materials and Methods

### 3.1. Materials

LM larvae were provided by Protifarm (Ermelo, The Netherlands) in freeze-dried form and stored at −20 °C before the analysis. Before each analysis, LM larvae were ground with an IKA A10 laboratory grinder for 2 min (IKA Werke GmbH & Co. KG, Staufen, Germany).

### 3.2. Total Amino Acids Profile

The total amino acid profile was evaluated according to the protocol proposed by Caligiani et al. [12] with some modifications. In particular, the analysis was performed by UPLC/ESI-MS using an ACQUITY UPLC separation system with an Acquity BEH C18 column (1.7 μm, 2.1 × 150 mm). The mobile phase was composed by H_2_O + 0.2% CH_3_CN + 0.1% HCOOH (eluent A) and CH_3_CN + 0.1% HCOOH (eluent B). Gradient elution was performed: isocratic 100% A for 7 min, from 100% A to 75.6% A and 24.4% B by linear gradient from 8 to 28 min, isocratic 100% B from 29 to 32 min, isocratic 100% A from 33 to 45 min. Flow rate was set at 0.25 mL/min, injection volume 2 μL, column temperature 35 °C, and sample temperature 18 °C. Detection was performed by using Waters SQ mass spectrometer: ESI source in positive ionisation mode, capillary voltage 3.2 kV, cone voltage 30 V, source temperature 150 °C, desolvation temperature 300 °C, cone gas flow (N_2_): 100 L/h, desolvation gas flow (N_2_): 650 L/h, full scan acquisition (270−518 m/z), scan duration 1 s. Calibration was performed with standard solutions prepared by mixing norleucine, amino acid hydrolysate standard mixture, cysteic acid, and deionised water. Tryptophan was determined as reported by Leni et al. [35]. The N-to protein conversion factor was calculated as described by Janssen et al. [20].

### 3.3. Lipid Extraction

Five g of finely ground LM larvae were defatted with Soxhlet fat extractor (SER 148/3 VELP SCIENTIFICA, Usmate Velate, Italy) and diethyl ether (60 mL) as extraction solvent. The sample was immersed in the boiling solvent for 60 min and subsequently subjected to a washing phase for 30 min and 10 min of solvent recovery.

### 3.4. Protein Extraction Protocols 

#### 3.4.1. One-Step Protein Extraction

A first chemical protocol was applied under mild conditions of alkali concentration as follows: a solution was prepared by combining 4 g of defatted LM larvae with 40 mL of 0.1 M NaOH and stirring using a magnetic stirrer at 50 °C for 2 h. Following extraction, the mixture was neutralised with 6M HCl. The resulting supernatant was separated from the pellet through centrifugation at 2683× *g* for 30 min at 4 °C (Centrifuge 5810/5810 R, Eppendorf, Milan, Italy). Both fractions were then stored at −20 °C for subsequent analyses.

#### 3.4.2. Stepwise Protein Extraction (Osborne Fractionation)

Protein extraction was also performed with an alternative chemical procedure based on the Osborne fractionation method, as described previously [12]. Briefly, 2 g of defatted sample were combined with 40 mL of 5 mM sodium ascorbate, 2 mM EDTA, and 10 mM Tris-HCl and stirred for 1 h at 4 °C. Subsequently, centrifugation at 2683× *g* for 20 min at 4 °C facilitated the separation of two fractions, with the supernatant collected as the albumin fraction. The remaining pellet underwent a similar process with 40 mL of 0.5 M NaCl, 5 mM sodium ascorbate, 2 mM EDTA, and 20 mM Tris-HCl, yielding the globulin fraction. After centrifugation, the pellet was mixed with 40 mL of 5 mM ascorbic acid in 70% EtOH, followed by centrifugation and collection of the prolamin fraction. The remaining pellet underwent extraction with 258 mL of 0.1 N NaOH and 5 mM ascorbic acid, producing the glutelin fraction. The 4 different protein fractions were collected and stored at −20 °C before the subsequent analyses.

#### 3.4.3. Enzymatic Protein Extraction

The extraction was carried out by employing protease from *Bacillus licheniformis* (PBL ≥ 2.4 U/g; EC Number 3.4.21.62; Merck, Darmstadt, Germany) at the optimal conditions for proteolysis, namely pH 7.5 and 60 °C, as suggested by the supplier. Specifically, 4 g of defatted LM larvae were mixed with 36 mL of a buffer solution (10 mM Na_2_HPO_4_, pH 7.5) and 40 µL of enzyme within a 100 mL reactor combined with a pH-STAT system and *tiamo* software (902 Titrando, Metrohm, Varese, Italy) that allowed to keep the pH stable during the reaction by gradually adding 0.2 M NaOH. The process began with hydrolysis conducted at a temperature of 60 °C for a duration of 6 h. Following this, the enzyme was rendered inactive by subjecting the solution to heat at 90 °C for 10 min. Subsequently, the hydrolysed sample underwent centrifugation at 2683× *g* for 30 min at a temperature of 4 °C, aimed at separating the supernatant from the pellet. Both fractions were then preserved at −20 °C to facilitate subsequent analyses.

##### Determination of the Degree of Hydrolysis (DH%)

The DH%, or degree of hydrolysis, is calculated as the proportion of peptide bonds that have undergone hydrolysis out of the total number of bonds within the sample and was calculated in the enzymatic hydrolysate by following Equation (1), as proposed by Butré et al. [39].

(1) $$DH\%=Vb\times Nb\times \frac{1}{\alpha }\times \frac{1}{htot}\times \frac{1}{Mp}$$

where ‘Vb’ is the volume (mL) of NaOH added by the pH-STAT system; ‘Nb’ is the normality (N) of NaOH; ‘α’ is the average degree of dissociation of the α-NH group; ‘Mp’ is the mass of protein (g) in the starting material; and ‘htot’ is the mmol of peptide bonds per g of protein substrate. ‘htot’ was calculated by dividing the mg of protein per gramme of substrate by 110, which represents the average molecular mass of the residual amino acid. α was calculated as described in Equation (2), while the pKa of the amino group was reported in Equation (3).

(2) $$\alpha =\frac{1}{1+10^{\left({pKa}_{{NH}_{2}}-pH\right)}}$$

(3) $${pKa}_{NH2}=7.8+\frac{\left(298-T\right)}{\left(298\times T\right)}\times 240$$

where T was the temperature expressed in Kelvin. Temperature and pH values were measured and recorded at regular time intervals of 10 s, to obtain a DH% value for each time point for the construction of the kinetic curve.

##### Free Amino Acid Determination by UPLC/ESI-MS

The free amino acid analysis was carried out on both LM larvae and enzymatic hydrolysate. In the first case, 0.5 g of ground LM were suspended in 5 mL of distilled water and mixed with 340 μL of 5 mM norleucine (dissolved in 0.1 M HCl) for 2 h. The volume was then adjusted to 10 mL with the addition of deionised water, and the solution was centrifuged for 30 min at 4 °C at 2683× *g*. For enzymatic hydrolysate, the sample was filtered on a 0.45 μm nylon filter membrane and collected. Moreover, 100 μL of filtrate were mixed with 34 μL of 5 mM norleucine (dissolved in 0.1 M HCl), and the volume was adjusted to 1 mL with deionised water. Then, 10 μL of the supernatant were derivatised with the AQC tag method and analysed with UPLC/ESI-MS, as described in Section 3.2. 

### 3.5. Determination of Protein Extraction Yield

The liquids obtained following both chemical and enzymatic extractions underwent Kjeldahl analysis using a DKL heating digestor and a UDK 139 semiautomatic distillation unit (VELP SCIENTIFICA). This analysis aimed to determine the percentage of nitrogen extracted. Protein extraction yield was calculated using the following Formula (4):

(4) $$Protein\;extraction\;yield=\frac{g\;of\;nitrogen\;in\;the\;supernatant}{g\;of\;total\;nitrogen\;before\;extraction}\times 100$$

Grammes of nitrogen in the supernatant were calculated taking into account the whole volume of extracts and assuming that it is completely from protein origin, since only a negligible amount derived from minor sources as nucleic acids, phospholipids, and ammonia. At the same time, the g of overall protein nitrogen prior to extraction were assessed by dividing the total amino acid content measured by LM with the appropriate conversion factor for nitrogen-to-protein.

### 3.6. Amino Acid Composition of the Extracts

The assessment of the nutritional potential of the isolated protein fractions, concerning their amino acid makeup, was conducted as outlined in Section 3.2 with certain adaptations. Initially, 3 mL of protein extract (protein content ranged from 0.5 to 5%) was combined with 3 mL of 12 M HCl to achieve a final concentration of 6 M for the hydrolysis process, which occurred at 110 °C for 23 h. Subsequently, 1 mL of 7 mM norleucine was added following the completion of hydrolysis, and the total volume was adjusted to 25 mL using deionised water. A portion of 100 μL from this solution was then mixed with 900 μL of 0.2 M borate buffer and subjected to AQC derivatisation prior to UPLC/ESI-MS analysis, following the procedure previously reported.

#### Determination of the Amino Acid Score of the Protein Fractions

The amino acid score of LM protein fractions was calculated by comparing the average values of each essential amino acid to the amino acid pattern of egg white. The amino acid score was calculated using the following Formula (5):

(5) $$Amino\;acid\;score=\frac{mg\;of\;AA\;in\;1\;g\;of\;protein}{mg\;of\;AA\;in\;1\;g\;of\;reference\;protein\;(egg\;white)}$$

The amino acid with the lowest score indicates the first limiting amino acid [40].

### 3.7. Statistical Analysis

All experiments were carried out in triplicate. Data are expressed as the mean ± standard deviation. The data were subjected to one-way analysis of variance (ANOVA) and Tukey’s post hoc test to determine the differences between samples. Statistical analysis was performed using SPSS version 21.0 (SPSS Inc., Chicago, IL, USA), and significant differences were compared at a level of *p* < 0.05.

## 4. Conclusions

LM represents a valuable source of alternative animal proteins, not only for the environmental and economic sustainability of their farming but also for the abundance of proteins with high nutritional quality. However, its consumption in western countries is considered taboo due to the absence of a history of entomophagy. A potential solution is represented by the incorporation of LM protein extracts as ingredients into common foods. The significance of this study is extended to the evaluation of the nutritional quality of diverse protein extracts, shedding light on the implications of varying extraction protocols on protein quality. LM larvae were rich in proteins (51% DM). As concerns their quality, the essential amino acids as a whole exceeded the requirement for human nutrition proposed by FAO, pointing out a great nutritional profile. Three extraction methods, including chemical (alkali extraction and Osborne stepwise fractionation) and enzymatic procedures (extraction by protease from *Bacillus licheniformis*), were carried out and compared for the protein extraction yield and amino acid composition. Results demonstrated how yield varied between 73% for the extraction performed with 0.1 M NaOH and 91.3% for the stepwise extraction, suggesting that alkali extraction alone could be insufficient to dissolve most proteins. The amino acid distribution of the extracts turned out to be scarcely affected by the extraction method from a nutritional point of view. However, the different distribution of some amino acids for the ‘stepwise’ protein fractions suggests potentially different technological applications. The protease-assisted extract showed a DH equal to 14%, indicating a partial breakdown of proteins into oligopeptides and free amino acids. These characteristics meet the growing demand for food ingredients with a reduced allergenicity risk and higher digestibility. Overall, the results indicate the possibility of modulating the composition of the LM protein fraction according to the specific application envisioned for the ingredient.

Future directions for research could involve further exploration of optimised extraction techniques and their effects on protein techno-functionalities, bioactivities, and sensory attributes. Additionally, further studies should investigate consumer perceptions and acceptance of products incorporating LM protein extracts.

## Figures and Tables

**Table 1:** Total amino acid content of LM larvae (expressed as g/100g DM and, for the essential amino acids, also as mg/g crude protein) compared to other food proteins and to the FAO standard protein.

	LM Protein (g/100g DM)	LM Protein (mg/g Protein)	Reference protein FAO/WHO 2013 (mg/g Protein)	Egg White (mg/g Protein)	Soybean (mg/g Protein) ^a^
**Essential AA**					
His	2.40 ± 0.01	41	15	23	25
Thr	2.58 ± 0.05	42	23	47	38
Val	3.61 ± 0.18	59	39	47	49
Lys	4.05 ± 0.11	68	45	65	63
Ile	2.60 ± 0.03	43	30	50	47
Leu	4.15 ± 0.05	69	59	81	85
Phe	2.97 ± 0.07				
Trp	1.22 ± 0.01	20	6		11
Met	1.25 ± 0.04				
Cys + Met		30 (9 + 21)	22	63 (24 + 39)	68
Phe + Tyr		130 (51 + 79)	38	93 (59 + 34)	97
**Non-essential AA**					
Asp + Asn	5.42 ± 0.01	90			
Ser	2.84 ± 0.05	45			
Glu + Gln	7.74 ± 0.03	130			
Gly	2.81 ± 0.01	41			
Arg	3.63 ± 0.18	62			
Ala	4.34 ± 0.11	66			
Pro	3.94 ± 0.08	64			
Tyr	4.57 ± 0.28				
Cys	0.66 ± 0.13				

Results are the means of triplicate analysis. ^a^ [22].

**Table 2:** Protein extraction yield obtained by different protocols applied to recover proteins from LM.

Protein Extraction Protocol		Protein Extraction Yield (%)
“One shot” chemical extraction	0.1M NaOH (2 h, 50 °C)	73 ± 3 ^a^
Enzymatic extraction	Protease from *Bacillus licheniformis*	76 ± 12 ^a^
“Stepwise” chemical extraction	Osborne fractionation	91.3 ± 0.2 ^b^
	ALBUMINS	30.8 ± 1.0
	GLOBULINS	2.6 ± 0.2
	PROLAMINS	5.5 ± 0.3
	GLUTELINS	52.4 ± 0.8

Results are the means of three separate extractions. Different letters in the same column indicate significant differences (*p* < 0.05).

**Table 3:** Amino acid score of lesser mealworms (LM) protein fractions calculated in comparison to the essential amino acid (EAA) profile of egg white.

EAA	LM	“One Shot” Extraction	“Stepwise” Fractionation				Enzymatic Hydrolysate
			Albumin	Globulin	Prolamin	Glutelin	
His	1.82	1.34	1.66	2.26	3.22	1.12	1.03
Ile	0.89	0.83	0.65	0.63	**0.39**	0.88	0.88
Leu	0.87	0.95	0.71	0.77	0.64	1.03	1.03
Met	**0.56**	**0.46**	**0.32**	**0.28**	0.51	**0.11**	**0.48**
Phe	0.88	0.83	0.62	0.85	0.92	0.82	0.84
Thr	0.92	0.86	0.97	1.05	0.81	1.07	1.12
Val	1.28	1.20	1.17	1.14	1.18	0.91	1.32
Lys	1.08	1.07	1.21	1.04	1.65	1.02	1.09
Tyr	2.39	2.33	1.33	2.36	2.10	2.47	2.43
Sum	1.09	1.04	0.90	1.03	1.11	1.01	1.10

The limiting amino acids are reported in bold.

**Table 4:** Free amino acid composition of lesser mealworm as such (LM) and of hydrolysate using commercial protease from *Bacillus licheniformis*.

Free Amino Acids (mg/g of Dry LM)	LM	Enzymatic Hydrolysate
Gly	0.18 ± 0.02 ^b^	5.3 ± 0.9 ^a^
Ala	0.8 ± 0.2 ^b^	19.9 ± 4.8 ^a^
Ser	0.21 ± 0.03 ^b^	5.0 ± 0.1 ^a^
Pro	1.4 ± 0.2 ^b^	14.5 ± 2.8 ^a^
Val	0.37 ± 0.03 ^b^	15.5 ± 3.7 ^a^
Thr	0.17 ± 0.03 ^b^	2.9 ± 0.3 ^a^
Ile	0.4 ± 0.2 ^b^	10.8 ± 2.8 ^a^
Leu	0.39 ± 0.05 ^b^	17.6 ± 3.6 ^a^
Asn	<0.1 ^b^	1.4 ± 0.2 ^a^
Asp	0.34 ± 0.08 ^b^	6.1 ± 1.1 ^a^
Gln	< 0.1 ^b^	0.9 ± 0.1 ^a^
Lys	0.10 ± 0.04 ^b^	17.5 ± 4.7 ^a^
Glu	1.0 ± 0.2 ^b^	22.0 ± 5.3 ^a^
Met	0.06 ± 0.01 ^b^	4.3 ± 1.1 ^a^
His	<0.1	<0.1
Phe	0.19 ± 0.01 ^b^	11.9 ± 4.3 ^a^
Arg	0.36 ± 0.07 ^b^	3.4 ± 0.9 ^a^
Tyr	0.30 ± 0.03 ^b^	2.0 ± 0.1 ^a^
Trp	0.16 ± 0.01 ^b^	5.6 ± 0.1 ^a^
Sum	6.4 ± 0.6 ^b^	166.7 ± 4.1 ^a^

Results are expressed as mg/g of dry insects employed for the analysis. Results are the mean of three separate experiments. Different letters in the same row show significant differences (*p* < 0.05).

## Data Availability

The data are contained within the article and Supplementary Materials.

## Supplementary Materials

The following supporting information can be downloaded at: https://www.mdpi.com/article/10.3390/molecules29174220/s1. Table S1: Amino acid distribution of LM and protein fractions.
