# Supplementary material for: Unravelling the Influence of Extraction Techniques on Protein Yield and Nutritional Value in Lesser Mealworm Larvae
Source: Molecules. 2024 Sep 5;29(17):4220. doi: 10.3390/molecules29174220 (PMC11397012; doi:10.3390/molecules29174220)
Supplement: Supplementary file 1 [file molecules-29-04220-s001.zip › molecules-3122942-supplementary.pdf]

Table S1 Amino acid distribution of lesser mealworm (LM) and protein fractions.

AA	LM	0.1M NaOH	Albumin	Globulin	Prolamin	Glutelin	PBL
Ala	68 ± 2 [ab]	75 ± 6 [bc]	85 ± 2 [c]	55 ± 5 [a]	57 ± 1 [a]	75 ± 2 [bc]	76 ± 3 [bc]
Asp/Asn	93 ± 0 [b]	101 ± 13 [bc]	90 ± 3 [b]	118 ± 3 [cd]	62 ± 2 [a]	124 ± 3 [d]	107 ± 3 [bcd]
Arg	64 ± 3 [a]	53 ± 11 [a]	46 ± 1 [a]	48 ± 5 [a]	95 ± 3 [b]	47 ± 3 [a]	47 ± 0 [a]
Gly	42 ± 0 [a]	46 ± 1 [abc]	51 ± 3 [c]	48 ± 0 [ab]	45 ± 3 [ab]	45 ± 2 [ab]	45 ± 0 [ab]
His	42 ± 0 [c]	31 ± 4 [ab]	38 ± 5 [bc]	52 ± 1 [d]	74 ± 2 [e]	26 ± 2 [a]	24 ± 1 [a]
Ile	44 ± 0 [b]	41 ± 0 [b]	32 ± 1 [ab]	32 ± 6 [ab]	20 ± 2 [a]	44 ± 1 [b]	44 ± 2 [b]
Leu	71 ± 1 [c]	77 ± 1 [cd]	57 ± 2 [a]	63 ± 0 [b]	52 ± 2 [ab]	83 ± 1 [d]	83 ± 1 [d]
Met	22 ± 1 [c]	18 ± 2 [bc]	12 ± 5 [ab]	11 ± 4 [ab]	20 ± 0 [c]	4 ± 0 [a]	19 ± 1 [bc]
Phe	52 ± 1 [b]	49 ± 7 [ab]	36 ± 4 [a]	50 ± 2 [ab]	54 ± 3 [b]	48 ± 0 [ab]	50 ± 2 [b]
Pro	66 ± 2 [b]	70 ± 1 [b]	118 ± 0 [d]	63 ± 3 [b]	102 ± 3 [c]	46 ± 0 [a]	51 ± 1 [a]
Ser	46 ± 1 [a]	52 ± 3 [a]	48 ± 1 [a]	50 ± 0 [a]	54 ± 5 [a]	51 ± 1 [a]	52 ± 2 [a]
Thr	43 ± 1 [abc]	41 ± 3 [ab]	45 ± 4 [abc]	49 ± 5 [bc]	38 ± 2 [a]	50 ± 1 [bc]	53 ± 1 [c]
Val	60 ± 3 [a]	57 ± 1 [a]	54 ± 3 [a]	54 ± 4 [a]	56 ± 4 [a]	43 ± 8 [a]	62 ± 2 [a]
Lys	70 ± 2 [a]	69 ± 9 [a]	85 ± 18 [a]	67 ± 7 [a]	108 ± 1 [b]	66 ± 0 [a]	71 ± 0 [a]
Tyr	81 ± 5 [b]	79 ± 12 [b]	45 ± 1 [a]	80 ± 4 [b]	71 ± 10 [ab]	84 ± 1 [b]	83 ± 0 [b]
Glu/Gln	134 ± 0 [b]	141 ± 14 [bc]	158 ± 3 [bc]	160 ± 2 [bc]	92 ± 10 [a]	163 ± 4 [c]	135 ± 3 [bc]

Results are expressed as mg AA/g of total protein and are the mean of three independent analyses. Different letters in the same raw indicate significant differences ($p<0.05$). PBL: Protease from B*acillus licheniformis*.
